# Analysis of the Optimal Duration of Behavioral Observations Based on an Automated Continuous Monitoring System in Tree Swallows (*Tachycineta bicolor*): Is One Hour Good Enough?

**DOI:** 10.1371/journal.pone.0141194

**Published:** 2015-11-11

**Authors:** Ádám Z. Lendvai, Çağlar Akçay, Jenny Q. Ouyang, Roslyn Dakin, Alice D. Domalik, Prianka S. St John, Mark Stanback, Ignacio T. Moore, Frances Bonier

**Affiliations:** 1 Department of Biological Sciences, Virginia Tech, Blacksburg, Virginia, United States of America; 2 Department of Evolutionary Zoology and Human Biology, University of Debrecen, Debrecen, Hungary; 3 The Netherlands Institute of Ecology (NIOO-KNAW), Wageningen, the Netherlands; 4 Department of Zoology, University of British Columbia, Vancouver, BC, Canada; 5 Department of Biology, Queen’s University, Kingston, ON, Canada; 6 Department of Biology, Davidson College, North Carolina, United States of America; Hungarian Academy of Sciences, HUNGARY

## Abstract

Studies of animal behavior often rely on human observation, which introduces a number of limitations on sampling. Recent developments in automated logging of behaviors make it possible to circumvent some of these problems. Once verified for efficacy and accuracy, these automated systems can be used to determine optimal sampling regimes for behavioral studies. Here, we used a radio-frequency identification (RFID) system to quantify parental effort in a bi-parental songbird species: the tree swallow (*Tachycineta bicolor*). We found that the accuracy of the RFID monitoring system was similar to that of video-recorded behavioral observations for quantifying parental visits. Using RFID monitoring, we also quantified the optimum duration of sampling periods for male and female parental effort by looking at the relationship between nest visit rates estimated from sampling periods with different durations and the total visit numbers for the day. The optimum sampling duration (the shortest observation time that explained the most variation in total daily visits per unit time) was 1h for both sexes. These results show that RFID and other automated technologies can be used to quantify behavior when human observation is constrained, and the information from these monitoring technologies can be useful for evaluating the efficacy of human observation methods.

## Introduction

The behavior of animals is notoriously variable. Therefore, finding a sampling regime that can accurately quantify behavior is challenging [1]. Most studies measuring animal behavior rely on human observation and subsequent analysis (‘coding’). However, regardless of whether the observer watches the animals directly or quantifies behavior from recorded video, the procedure requires considerable time and effort. Consequently, availability of human resources and/or video recording equipment limits such studies of animal behavior. In addition, it may be desirable to limit disturbance of the animals, (e.g., to reduce impacts of the observer on behavior), further constraining human activity around the study subjects. Even if there were no limits or constraints on human observation, statistical power rises as an asymptotic function of sample size; thus, after a certain point, the value of each additional sample begins to decline. Therefore, it may be more efficient to stop data collection before the informational asymptote is reached, to maximize the return for observer effort [2]. For all these reasons, a careful consideration of sampling effort is warranted.

Although the duration of observation periods has important consequences for statistical power, and thus the required sample size and effort, often the duration of observation periods used in a given study seems arbitrary. For instance, many behavioral studies of parental behavior use 1 hour behavioral watches [3–5], or sometimes even shorter observation periods [6–10]. These studies do not explicitly justify or validate the duration of the chosen observation period; therefore, the degree to which these observational samples are representative of subjects’ behavior on longer time-scales is often unknown. Although several studies have provided analyses of different sampling regimes [2,11–13], these results may be difficult to generalize across species because of potential differences in the nature of behavior. Furthermore, some of these studies have relied solely on direct observations, which are by definition limited by manpower and human attention (e.g., a human observer cannot reasonably watch focal individuals from dawn to dusk), and human presence may also alter the behavior being studied.

Here, we use continuous recordings of parental provisioning visits from two populations of tree swallows (*Tachycineta bicolor*) to investigate the effect of different behavioral observation sample durations on the accuracy of estimated provisioning rates. We used an automated monitoring system based on radiofrequency identification (RFID) technology [14] that recorded every visit of the parents to the nest box throughout the entire day. Our aims were to determine the effect of observation period duration and statistical accuracy of estimated visit rate, so we can aid other researchers in choosing a sampling regime for their particular study system, and to demonstrate the degree to which duration of sampling regime can influence accuracy. We first validated RFID readings with data from 1-hr behavioral observations. Next, we estimated the optimal duration of behavioral observations that would maximize the amount of between-nest variation in parental behavior explained, while minimizing the effort to collect such samples. In doing so, we also emphasize that the optimal observation period for other systems may differ depending on various factors which we discuss below. Nonetheless, our approach to estimating the relationship between sampling effort and proportion of variance explained could be used in other systems to determine the required sampling effort to obtain a desired degree of accuracy.

## Materials and Methods

### Study populations

We investigated nestling provisioning behavior in a bi-parental songbird, the tree swallow, in two populations: at the Queen’s University Biological Station, Ontario, Canada (N44°34’2.02”, W76°19’26.036”, 121m elevation) in 2014, and near Davidson College, Davidson, North Carolina, USA (N34°31’ 32.34”, W80°52’40”, 240m elevation) in 2014 and 2015. All procedures followed guidelines for animal care outlined by Association for the Study of Animal Behaviour, and the Animal Behavior Society and the Canadian Council on Animal Care, and were approved by the Institutional Animal Care and Use Committee at Virginia Tech (#12–020) and the Canadian Wildlife Service (#10771). In both populations, birds breed in nest boxes [15,16]. In tree swallows, females feed their offspring at a higher rate than males on average [17], and male visit rates show higher among-individual variance than female visit rates (RD, JQO, AZL unpublished data).

### Bird tagging and data collection

Both parents were captured in their nest box (females: day 10 of incubation, males: day 2 or 3 post hatching) and equipped with a PIT-tag (passive integrated transponder) that was incorporated into a plastic leg band (EM4102 tags from IB Technology, UK). These leg bands were red for females and blue for the males. A hexagonal or square antenna (diagonally about 6cm) was fixed around the entrance of the nest box, which was later (from day 3 to day 5 post hatching), connected to an RFID reader. The reader attempted to detect a signal for 0.3 seconds, then paused for 0.2 seconds to save battery life and then this cycle was repeated continuously. This way, the reader recorded every time a bird equipped with a PIT tag passed through the antenna and thus the nest box entrance. The reader recorded the unique tag number and the current date and time to the seconds in a log file. We used “Generation 2” readers, an upgrade of the model described in [18] provided by Cellular Tracking Technology, PA, USA. The readers were powered from a 12V, 5Ah motorcycle battery (8.9×7.1×10.1 cm). The reader and the battery were placed in a waterproof plastic container and hidden in the grass, below the nest box. To save power, we programmed the readers to turn off during the night (between 22:00 and 04:00). Therefore, on day 5, the readers recorded all visits that either parent made to the box during the entire day at n = 18 nests. In 46 cases, the readers were first set up on day 5, typically in the morning, between 07:00 and 10:00, so the duration of daily recordings is shorter for these nests, but still covers most of the day (mean: 12.72 ± 0.18 (SE) hours at a site with approximately 15 hours of daylight). In an additional 10 nests, RFID readers were deployed in the same manner, but the RFID readers yielded fewer than 200 total reads for that day (male and female combined; compared to the rest of the nests, where the average number of total reads was 1281 ± 149 (SE)), which indicates that the tags or antennae at these nests were not working properly, or that the parents fed their nestlings at an unusually low rate. These nests were excluded from our analyses. The final sample sizes for RFID analyses in 2014 were 34 (Canada) and 30 (US) nests. To test whether our conclusions can be generalized through a wider range of nestling ages, in 2015, we also collected RFID logs from 13 nests on day 3 post hatching and 28 nests day 8 post hatching (US only).

From the RFID logs, we determined the number of nest visits by filtering out continuous readings, generated when a bird is perching on the nest entrance (i.e., adjacent to the antenna). Our measure of visit rate based on the RFID logs may overestimate the actual number of feeding visits (e.g., birds sometimes go into the nest box, reappear at the entrance and then go back to the box before finally leaving the box–this event would be treated as two separate visits in our analyses). Such cases, however, were relatively infrequent (see Results).

In 2014, each nest was also directly monitored by a human observer for one hour to quantify the visit rates of the parents, and to determine whether RFID logs provide a similar estimate of visit rates by correlating the observational data with the visit rate calculated from the RFID logs. A total of 45 nests were directly observed while the RFID readers were in operation. The observer sat at about 30 m from the nest box at an angle that would allow him or her to determine the color of band (and therefore the sex) every time a bird entered. Because our primary interest in this study was accuracy in quantifying between-nest variation, we used only one day (day 5) of observation at a standard stage of chick rearing.

### Statistical analyses

Our analyses proceeded in two stages. In the first stage, we compared the visits inferred from the RFID logs with the visits noted during the observations for the same hour. In the second stage of our analyses, we used the RFID data to determine if different sampling durations could reliably estimate overall daily behavior. We first calculated the overall daily visit rate (number of visits divided by the duration of the total recording period) for both males and females in each nest from the RFID logs. We used the same logs and sampled 1h-long periods starting at different times of the day using all possible start times and calculated the sample visit rate again for both sexes. Then, separately for males and females, we used a linear regression to test how well visit rates calculated from the 1h samples predict the total daily visit rates. Because our focus was on between-nest variation, we extracted the R^2^ from the linear model as a measure of the proportion of variance explained. We also obtained 95% confidence intervals for these estimates using nonparametric bootstrapping. Specifically, we calculated the R^2^ of the linear relationship between the hourly and the daily feeding rate using a random sample with replacement and 10000 replicates.

Next, we repeated the above process while varying the duration of the sampling window from 15min to 4h by 15-min increments. We set the maximum at 4h because, in most field conditions, longer direct observations are not feasible, and even with video recordings, sampling is constrained by battery. For every hour from 07:00 to 17:00, we calculated the R^2^ based on different sampling window durations separately for the sexes.

We next sought to determine the optimal sampling duration. To do that, we first fit a series of curves to the R^2^ obtained at different observation periods. We fitted multiple curves because, while we expected the data would follow a saturation curve (i.e., very long observations will reach an asymptote in terms of proportion of between-individual variation explained), we did not have an a priori expectation that the data would fit one particular type of saturation curve over another. In practice, the fitted curves differed little in their shape (see Results). We fit three models that are often used to model such relationships, using the package ‘drc’ [19] in the R computing environment (version 3.2) [20]. First, we fitted a three-parameter Gompertz growth curve. The Gompertz curve converges towards an asymptote and the steepness of the curve changes with an inflection point in between the start and the asymptotic part of the curve. Next, we fitted a three parameter Michaelis-Menten model, a saturation curve that does not have an inflection point, and a three parameter asymptotic regression. We estimated the goodness of fit of each model using ‘modelFit’ in ‘drc’, where a significant value indicates a lack of fit, and used the second order Akaike Information Criterion to compare the fit of different models. Finally, we also fit a general additive model to the data using the ‘gam’ function in the ‘gam’ package that uses penalized regression splines. This method fits the model using a penalized likelihood maximization, in which the model likelihood is modified by the addition of a penalty for each smooth function, resulting in a balance between smoothness and goodness of fit. It does not assume that there is an inflection point or asymptote.

We then used two optimization algorithms to find the marginal value that gives the optimal sampling effort, defined as the one that maximizes the rate of return of statistical accuracy in R^2^ units per unit of sampling time. First, for the Gompertz fit, we took the local minimum of the second derivative of the fitted curve, which gives the inflection point of the first derivative where the concavity of the steepness of the curve changes towards the asymptotic decrease. For the other fits, the steepness of the curve monotonically decreases, and therefore there is no inflection point. In these cases we used the ‘minimally important change’ threshold that is often used in clinical trials to find a balance between specificity and sensitivity of a treatment (that also follows a hyperbolic saturation curve), and that has been recently shown to provide the optimal cutoff value [21]. This method uses a sum of squares method to find the point on the curve that maximizes the outcome while minimizing the cost (in our case, statistical accuracy and observational duration, respectively). An R script of the analyses (S1 File) and the dataset (S2 File) are provided as electronic supporting information.

## Results

**Fig 1:**
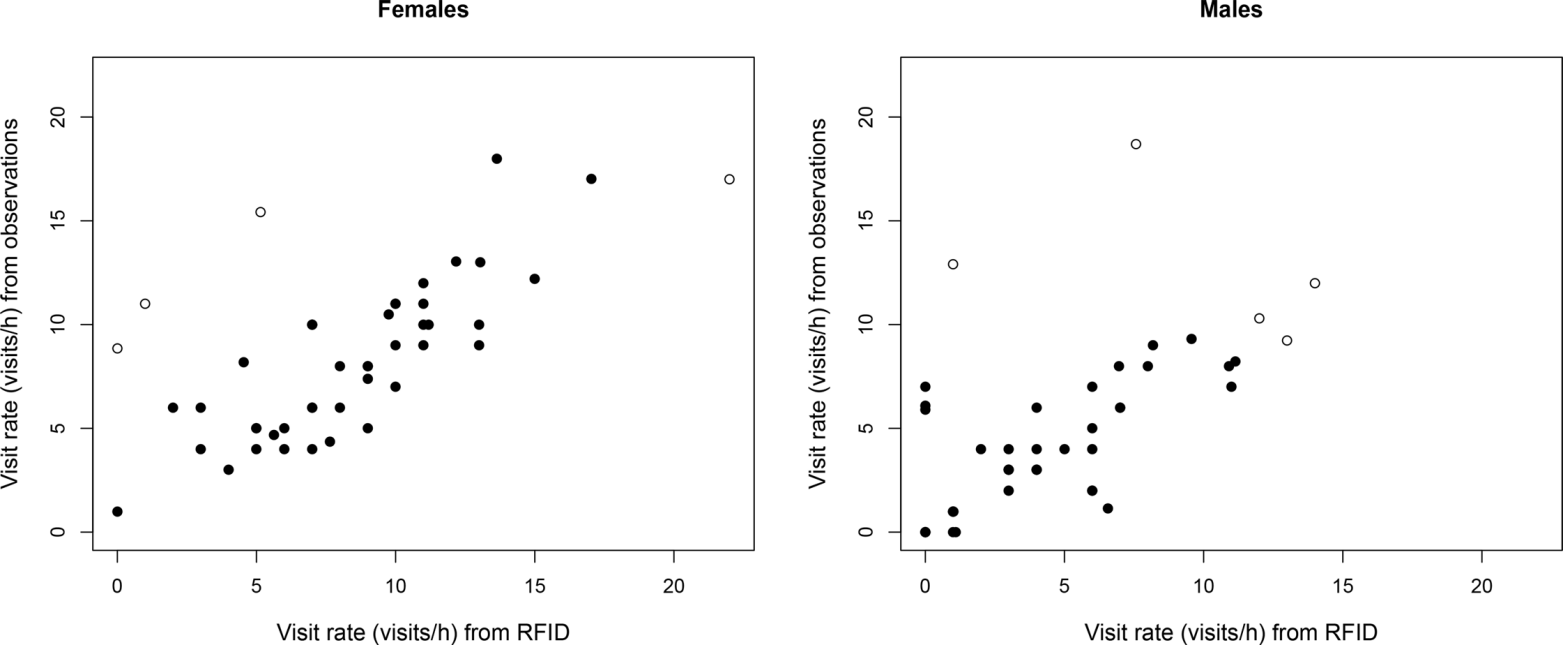
Visit rate (the number of feeding visits/h) of female and male tree swallows inferred from 1h-behavioral observations (y-axis) and RFID readings (x-axis). Open circles denote influential data points that have disproportionate effect on the relationship as measured by the ‘influence.measures’ function in R. Note that the statistical analyses provided in the main text were carried out including these data points, and therefore provide a conservative estimate of these relationships.

Visit rates calculated from day 5 RFID logs and direct behavioral observations were highly correlated (females: r = 0.68, p = 0.2 × 10^−7^ and males: r = 0. 67, p = 0.4 × 10^−7^; N = 43, Fig 1). There was a strong positive linear relationship between visits inferred from RFID logs and directly observed visits, with only a few exceptions (Fig 1). In most cases, the exceptions involved the failure of the RFID system to detect visits that were noted by an observer, which may have been due to failure of the PIT-tag or the antenna, although observer error is also possible.

**Fig 2:**
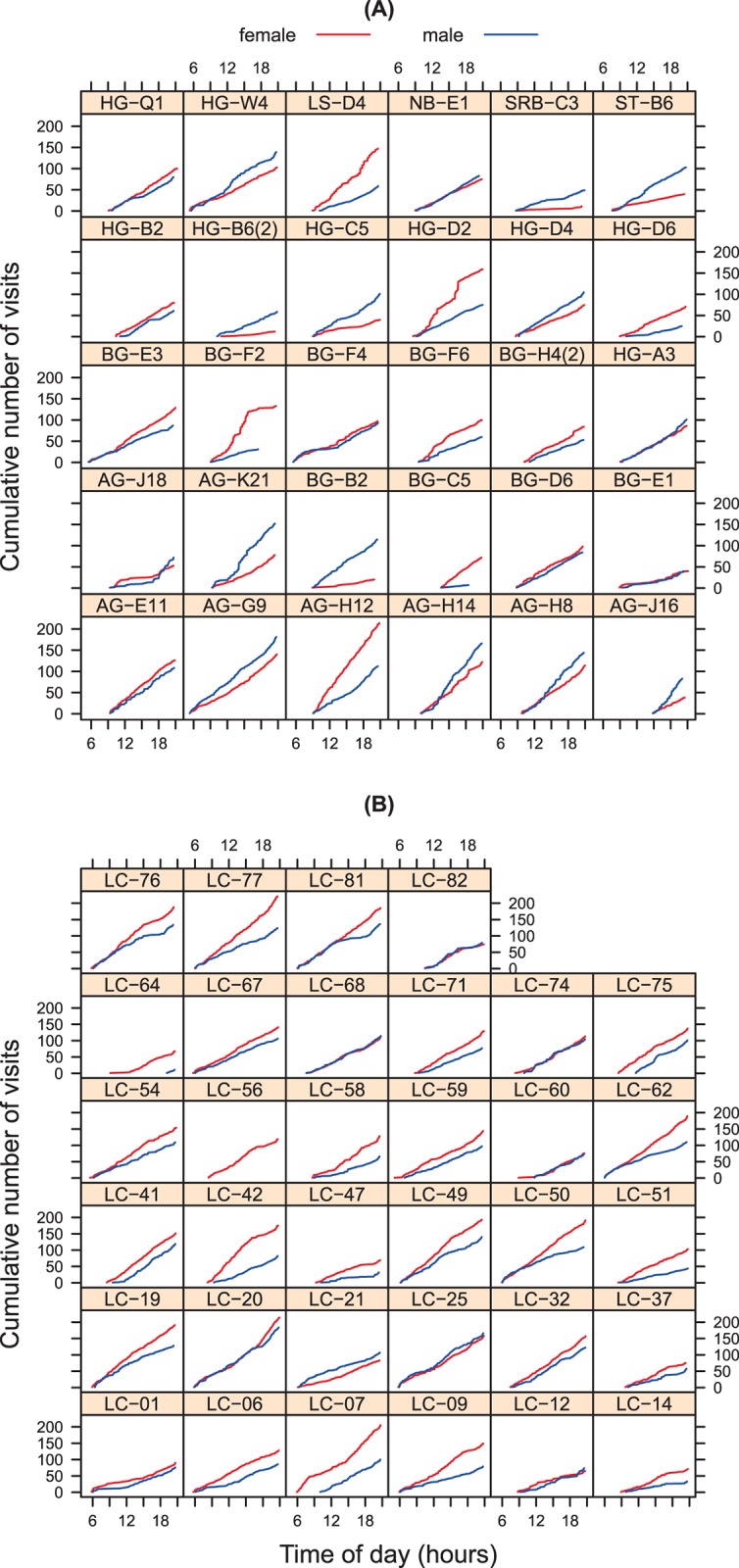
The cumulative number of parental visits in tree swallow nests in (A) Canada and (B) North-Carolina. In both (A) and (B), each panel corresponds to one nest (the nest identifier is printed above each panel), with the blue line representing the male and the red line the female parent.

Next, we looked at the RFID logs of the entire day. In most nests, the cumulative number of visits increased monotonically and linearly during the day in both sexes (Fig 2), suggesting that diel variation in visit rate was negligible.

After combining data from both populations, we examined how the time of day when the 1h sample began predicted the total daily visit rate. Observations of 1h in duration significantly predicted the total daily visit rate across all start times (Table 1). However, the proportion of variance explained depended on when the 1h sampling began. Mid-day sampling tended to provide the best estimates, whereas evening and early morning hours gave the worst estimates for both females and males.

**Table 1:** Proportion of variance explained (R^2^) and its 95% confidence interval generated by bootstrapping, statistical significance (p-values), and the sample size (N) of the relationship between 1h-samples and the total daily visit rate based on the time of onset of the 1h-sample for female and male tree swallows.

time	R^2^ [95% CI] (female)	p-value (female)	R^2^ [95% CI] (male)	p-value (male)	N
06:00	0.60 [0.31; 0.83]	4.3e-04	0.34 [0.08; 0.68]	1.9e-02	16
07:00	0.57 [0.41; 0.77]	3.2e-04	0.26 [0.05; 0.57]	3.0e-02	18
08:00	0.40 [0.15; 0.77]	2.0e-03	0.24 [0.04; 0.54]	2.5e-02	21
09:00	0.40 [0.19; 0.60]	9.2e-06	0.36 [0.18; 0.57]	3.5e-05	41
10:00	0.52 [0.31; 0.70]	4.8e-10	0.25 [0.09; 0.43]	1.2e-04	55
11:00	0.59 [0.42; 0.74]	2.9e-13	0.16 [0.03; 0.41]	1.1e-03	62
12:00	0.66 [0.52; 0.78]	1.6e-15	0.52 [0.33; 0.71]	4.0e-11	62
13:00	0.70 [0.55; 0.81]	2.0e-16	0.65 [0.46; 0.78]	2.2e-15	62
14:00	0.53 [0.35; 0.71]	1.8e-11	0.47 [0.28; 0.63]	5.6e-10	63
15:00	0.50 [0.31; 0.68]	6.0e-11	0.64 [0.46; 0.77]	2.7e-15	64
16:00	0.31 [0.20; 0.59]	1.6e-06	0.30 [0.13; 0.48]	3.0e-06	64
17:00	0.49 [0.31; 0.66]	1.4e-10	0.50 [0.28; 0.68]	8.4e-11	64
18:00	0.44 [0.25; 0.61]	3.1e-09	0.59 [0.35; 0.81]	8.9e-14	64
19:00	0.50 [0.30; 0.66]	8.7e-11	0.47 [0.27; 0.64]	3.9e-10	64

**Fig 3:**
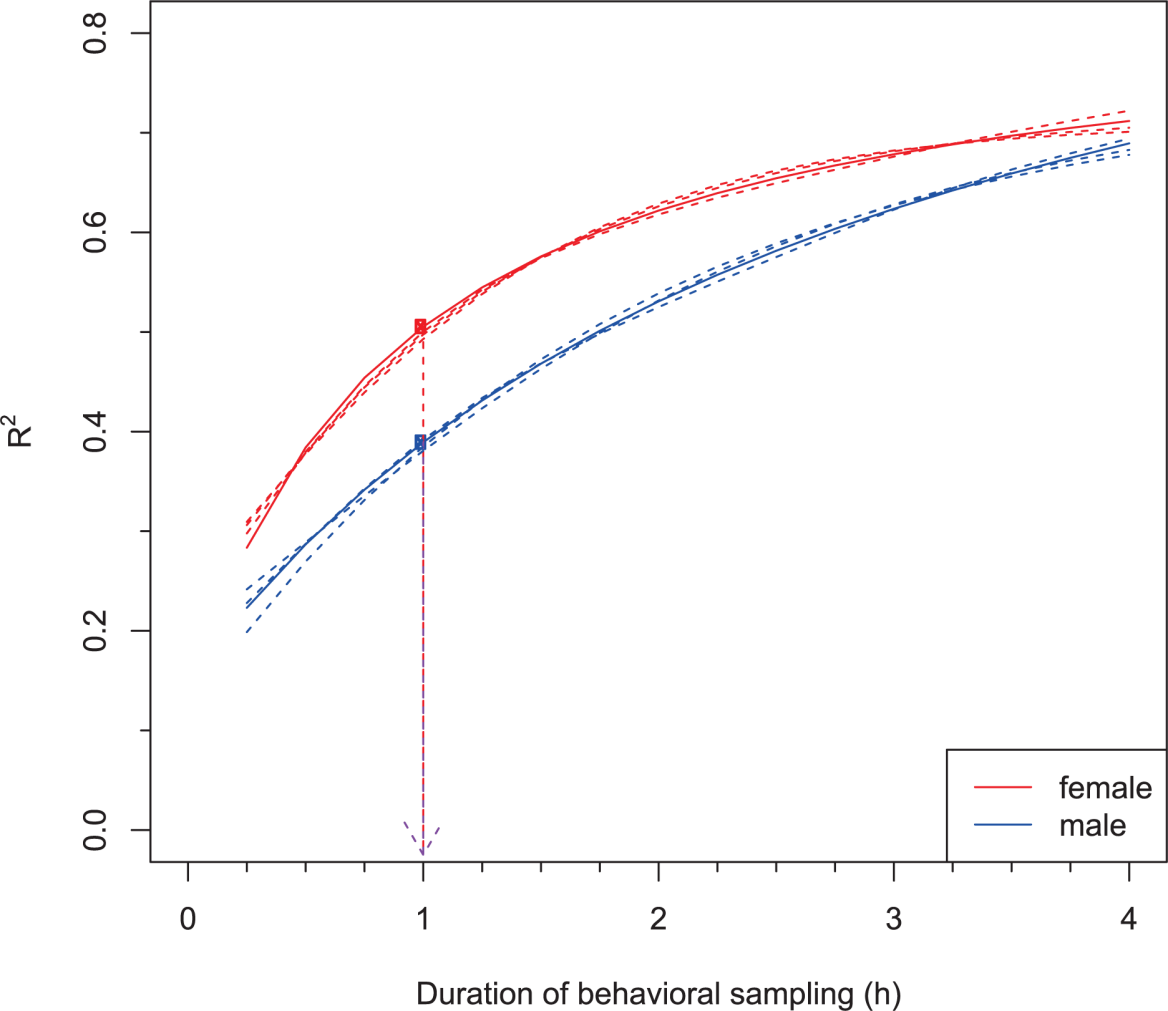
Optimal durations of observation periods for female and male tree swallows. The solid lines show the best fit curve to the data (a three parameter Michaelis-Menten model) for the relation between R^2^ and observation period duration (15 minutes—4 hours). The dashed lines show three alternative model fits (Gompertz, Asymptotic regression and General Additive Model). Red and blue dots indicate the optimal sampling effort for females and males respectively, that maximizes R^2^ and minimizes the duration of observation (indicated by the dashed arrows).

All of the parametric models we tested showed good fit to the data with the monotonic Michaelis-Menten model giving the best fit for both sexes (females: F = 0.078, p = 1.0, males: F = 0.036, p = 1.0). The Gompertz and the asymptotic regression (AR) models showed similar fit, but were somewhat less supported for the female dataset (ΔAICc = 3.763 and 1.90 for Gompertz and AR respectively), whereas for the male dataset the difference was even smaller (ΔAICc = 0.860 and 1.116, respectively), therefore these alternative models explained the relationship between duration of observation and R^2^ equally well (Fig 3). The general additive model (GAM) provided a monotonic smooth curve for both males and females, but these models had the least support (females: ΔAICc = 7.05, males: ΔAICc = 3.33).

Despite these differences in model fit, the Euclidean optimization function provided the same optimal duration for observations for all 4 curves, with an estimate of 1h for both sexes (Fig 3). The concavity approach based on the Gompertz curve provided optimal duration estimates of 45 minutes for females and 1.5 hours for males.

Repeating the same analyses on day 3 and day 8 logs on a different set of individuals from 2015 gave identical results. The optimal duration of sampling (calculated using the Euclidean optimization) was 1h for both males and females provisioning younger (day 3) and older (day 8) nestlings. Similarly to the day 5 records, the concavity approach provided estimates of 45 minutes for females and 1.5 hours for males as an optimal duration for both day 3 and day 8 nestling ages.

## Discussion

In this study, we demonstrated the utility of RFID data loggers for quantifying nest visit rates in a small songbird, and quantified the relationship between sampling period duration and statistical accuracy of estimates of parental behavior. We provide an optimization method that can be easily applied to provisioning data from other systems, whether collected by behavioral observations or by an automated recording system. Our results therefore provide a template for other behavioral studies seeking to measure behavioral traits with accuracy while maximizing efficiency.

For chick-rearing tree swallows, the optimal sampling period duration of about 1h for both sexes was robust to different curved fits to the data. A different optimization algorithm based on the change of the steepness of the curve provided a slightly different estimate: 45 min for females and 1.5h for males. Note that the latter approach only works with the Gompertz growth function with an inflection point. The Gompertz function did not fit our data as well as the monotonic Michaelis-Menten function, although the differences between these fits were small (Fig 3). We recommend using the ‘minimally important change’ threshold [21] that uses simple Euclidean geometry and works with all presented model fits. This method is widely used in the medical fields [21], but has not been applied in an ecological context. We provide a script to perform this analysis as an electronic supplement (S1 File), so that other researchers can apply it to find the optimal sampling duration for their study systems.

Our data suggest that, depending on whether researchers want to analyze females, males, or both sexes, observation periods of between 45 and 90 minutes are ideal for a study of tree swallow parental feeding rates. Although the feeding rate of the parents may change as the nestlings grow (e.g., [22,23] but see [24]), nestling age had no effect on the optimal sample duration. This conclusion seems to corroborate a growing list of studies that tested whether shorter observation durations can predict the parental behavior measured from a longer, whole-day sample [13]. These studies often conclude that 1h observation is sufficient to reliably reflect the variation in feeding rates among individuals (Table 2). These studies, however, typically tested only 1h or 2h as a sampling period. Here, we tested 16 different sample durations (from 15 mins to 4h) across the entire day. We found that 1h was in fact the optimal sampling time, given that it maximized accuracy while minimizing total sampling effort.

**Table 2:** Summary of published results testing different sampling regimes.

Species	Data collection method	Sampling durations	Is 1h good enough?^a^	Reference
Eastern kingbird (*Tyrannus tyrannus)*	observations	1h *vs* 2–3h	yes	[13]
Savannah sparrow (*Passerculus sandwichensis*)	observations	2h *vs* whole day	1h was not tested, but 2h samples gave estimates that agreed closely with the longer observations	[25]
Blue tit (*Cyanistes caeruleus*)	RFID	1h or 2h *vs* whole day	yes	[26]
Blue tit (*Cyanistes caeruleus*)	RFID	1h *vs* whole day	yes	[11]
House sparrow (*Passer domesticus*)	observations	1h or 2h *vs* whole day	yes, but 2×1h or 2h observations yielded more accurate estimates	[2]
Great tit (*Parus major*)	infrared microcamera	1h *vs* 7h (7:00–14:00)	yes	[12]
Tree swallow (*Tachycineta bicolor*)	RFID	15 min- 4h *vs* whole day	yes	this study

^a^ This column indicates whether 1h sample could significantly predict longer (or whole day) provisioning behavior.

Interestingly, we did not observe a systematic effect of time of the day on accuracy (R^2^), although early morning and evening samples tended to give poorer estimates. Indeed, the cumulative number of observations increases steadily throughout the day in a linear fashion, which is consistent with earlier observations that tree swallows feed their young during daylight hours at a relatively constant rate [27,28]. Studies of avian parental care usually concentrate on the morning hours, mainly because the activity of insectivorous birds is often the highest during the early hours of the day and one might think that a relatively short observation period is the most reliable when there are a lot of behavioral activities to record. However, our results corroborate earlier conclusions that this is not necessarily the case [11]. For example, in the blue tit (*Cyanistes caeruleus*), parental feeding rate is indeed the highest in early morning. However, the sex differences in blue tit feeding rates are also greater during the early hours, therefore, sampling these birds only during these hours could provide an inflated and the least reliable estimate of variation in sex differences in parental care patterns of this species [11].

We emphasize, however, that our approach here has been purely pragmatic, and increasing observation period duration to be greater than 1h will always yield greater accuracy. If sample size is low, this may be desirable to attain greater statistical power. In our dataset, an increase of observation period duration from 1h to 2h could explain an additional ~15% of the variance (Fig 3). So, as always in optimization, the currency will determine the optimal approach. We believe that being able to quantify the gains of increased sampling periods, as we do here, will be valuable to researchers trying to find the optimum sampling regime for their own system. But researchers also need to consider minimum level of variation explained that would be acceptable for their study, as well as other, e.g., logistical, constraints.

Finally, our data validate the use of RFID technology as a powerful tool to estimate parental visit rates. This tool provides an effective method for behavioral ecologists to circumvent the logistical and human resource limitations and observation bias that researchers face when designing behavioral field studies [29]. It is important to note that the RFID readers cannot discriminate between different behaviors performed during visits (such as feeding, brooding, nest defense, or courtship/copulation), and as such these methods are not yet able to completely replace behavioral observations for a variety of scientific questions (e.g. when researchers are interested in classifying types of social interactions). That said, the benefits of all-day monitoring might outweigh the limitations of such a system, for some scientific questions, such as those that require quantification of feeding rates in nestbox breeding birds. Furthermore, the results presented here will be useful to those researchers using only behavioral observations as well. We believe the combination of behavioral observations with RFID (or similar) monitoring technologies is the most fruitful strategy for field research in the immediate future.

## Supporting Information

S1 File R script of the analysis. (R)

S2 File Parental feeding rates by 15 minutes intervals provided as an R dataset. (RDATA)
